# Supplementary material for: Effectiveness of Tri-Kaysorn-Mas Extract in Ameliorating Cognitive-like Behavior Deficits in Ovariectomized Mice via Activation of Multiple Mechanisms
Source: Pharmaceuticals (Basel). 2024 Sep 8;17(9):1182. doi: 10.3390/ph17091182 (PMC11435318; doi:10.3390/ph17091182)
Supplement: Supplementary file 1 [file pharmaceuticals-17-01182-s001.zip › pharmaceuticals-3101788-supplementary.pdf]

Supplementary Information (SI)

Effectiveness of Tri-Kaysorn-Mas Extract in Ameliorating Cognitive-like Behavior Deficits in Ovariectomized Mice via Activation of Multiple Mechanisms

Abdulwaris Mading[1,2], Yutthana Chotritthirong[1,2], Yaowared Chulikhit[2], Supawadee Daodee[2], Chantana Boonyarat[2], Charinya Khamphukdee[3], Wanida Sukketsiri[4], Pakakrong Kwankhao[5], Supaporn Pitiporn[5] and Orawan Monthakantirat[2,*]

1 Graduate School of Pharmaceutical Sciences, Khon Kaen University, Khon Kaen, 40002, Thailand; abdulwaris.m@kkumail.com (A.M.); yutthana_ch@kkumail.com (Y.Ch.)

2 Division of Pharmaceutical Chemistry, Faculty of Pharmaceutical Sciences, Khon Kaen University, Khon Kaen, 40002, Thailand; yaosum@kku.ac.th (Y.C.); csupawad@kku.ac.th (S.D.); chaboo@kku.ac.th (C.B.); oramon@kku.ac.th (O.M.)

3 Division of Pharmacognosy and Toxicology, Faculty of Pharmaceutical Sciences, Kaen University, Khon Kaen, 40002, Thailand; charkh@kku.ac.th (C.K.)

4 Division of Health and Applied Sciences, Faculty of Science, Prince of Songkla University, Hat Yai, Songkhla, 90110, Thailand; wanida.su@psu.ac.th (W.S.)

5 Department of Pharmacy, Chao Phya Abhaibhubejhr Hospital, Ministry of Public Health, Prachinburi, Thailand 25000, Thailand; pakakrong2@gmail.com (P.K.); spitiporn@yahoo.com (S.P.)

* Correspondence: oramon@kku.ac.th; Tel.: +66-804265989

1. Statistical Analysis Effect of TKM Extract on OVX-Induced Cognitive-Like Behavior using Y-Maze Test.

Table S1.1 One-way analysis of variance (ANOVA) test of the percentage of alternation.

**Group comparison**	**Statistical analysis**	
	P	**F ($DF_{between\ group}$, $DF_{residual}$)**
ANOVA followed by Tukey's post hoc test		F (4,45) = 28.982
All group	< 0.001	
Sham vs. OVX + vehicle group	< 0.001	
OVX + vehicle group vs. OVX + 17β-estradiol	< 0.001	
OVX + vehicle group vs. OVX + TKM50	0.908	
OVX + vehicle group vs. OVX + TKM100	< 0.001	
OVX + TKM50 vs. OVX + TKM100	< 0.001	

Table S1.2 One-way analysis of variance (ANOVA) test of Locomotor test.

**Group comparison**	**Statistical analysis**	
	P	**F ($DF_{between\ group}$, $DF_{residual}$)**
ANOVA followed by Tukey's post hoc test		F (4,45) = 1.256
All group	0.301	
Sham vs. OVX + vehicle group	0.532	
OVX + vehicle group vs. OVX + 17β-estradiol	0.946	
OVX + vehicle group vs. OVX + TKM50	0.770	
OVX + vehicle group vs. OVX + TKM100	0.999	

2. Statistical Analysis Effect of TKM Extract on OVX-Induced Cognitive-Like Behavior using Novel Object Recognition Test (NORT).

Table S2 One-way analysis of variance (ANOVA) test of NORT.

**Group comparison**	**Statistical analysis**	
	P	**F ($DF_{between\ group}$, $DF_{residual}$)**
ANOVA followed by Tukey's post hoc test		F (4,45) = 17.163
All group	< 0.001	
Sham vs. OVX + vehicle group	< 0.001	
OVX + vehicle group vs. OVX + 17β-estradiol	< 0.001	
OVX + vehicle group vs. OVX + TKM50	0.908	
OVX + vehicle group vs. OVX + TKM100	< 0.001	
OVX + TKM50 vs. OVX + TKM100	0.013	

3. Statistical Analysis Effect of TKM Extract on OVX-Induced Cognitive-Like Behavior using Morris Water Maze test (MWM).

Table S3.1 One-way repeated measurement ANOVA test of MWM on the training phase

**Day 1**		
**Group comparison**	**Statistical analysis**	
	P	**F ($DF_{between\ group}$, $DF_{residual}$)**
ANOVA followed by Tukey's post hoc test		F (4,45) = 1.342
All group	0.269	
Sham vs. OVX + vehicle group	0.598	
OVX + vehicle group vs. OVX + 17β-estradiol (E2)	0.648	
OVX + vehicle group vs. OVX + TKM50	1.000	
OVX + vehicle group vs. OVX + TKM100	1.000	

Day 2		
**Group comparison**	**Statistical analysis**	
	P	**F ($DF_{between\ group}$, $DF_{residual}$)**
ANOVA followed by Tukey's post hoc test		F (4,45) = 1.876
All group	0.131	
Sham vs. OVX + vehicle group	0.065	
OVX + vehicle group vs. OVX + 17β-estradiol (E2)	0.560	
OVX + vehicle group vs. OVX + TKM50	0.684	
OVX + vehicle group vs. OVX + TKM100	0.661	
**Day 3**		
**Group comparison**	**Statistical analysis**	
	P	**F ($DF_{between\ group}$, $DF_{residual}$)**
ANOVA followed by Tukey's post hoc test		F (4,45) = 3.858
All group	0.009	
Sham vs. OVX + vehicle group	0.003	
OVX + vehicle group vs. OVX + 17β-estradiol (E2)	0.132	
OVX + vehicle group vs. OVX + TKM50	0.179	
OVX + vehicle group vs. OVX + TKM100	0.119	
**Day 4**		
**Group comparison**	**Statistical analysis**	
	P	**F ($DF_{between\ group}$, $DF_{residual}$)**
ANOVA followed by Tukey's post hoc test		F (4,45) = 3.876
All group	0.009	
Sham vs. OVX + vehicle group	0.005	
OVX + vehicle group vs. OVX + 17β-estradiol (E2)	0.063	
OVX + vehicle group vs. OVX + TKM50	0.101	
OVX + vehicle group vs. OVX + TKM100	0.067	
**Day 5**		
**Group comparison**	**Statistical analysis**	
	P	**F ($DF_{between\ group}$, $DF_{residual}$)**
ANOVA followed by Tukey's post hoc test		F (4,45) = 4.278
All group	0.005	
Sham vs. OVX + vehicle group	0.003	
OVX + vehicle group vs. OVX + 17β-estradiol (E2)	0.084	
OVX + vehicle group vs. OVX + TKM50	0.040	
OVX + vehicle group vs. OVX + TKM100	0.037	

Table S3.2 One-way analysis of variance (ANOVA) test of MWM on probe test.

Group comparison	Statistical analysis	
	P	**F ($DF_{between\ group}$, $DF_{residual}$)**
ANOVA followed by Tukey's post hoc test		F (4,45) = 70.104
All group	< 0.001	
Sham vs. OVX + vehicle group	< 0.001	
OVX + vehicle group vs. OVX + 17β-estradiol	< 0.001	
OVX + vehicle group vs. OVX + TKM50	0.439	
OVX + vehicle group vs. OVX + TKM100	< 0.001	
OVX + TKM50 vs. OVX + TKM100	< 0.001	

4. *Statistical Analysis Effect of TKM Extract on OVX-Changed Serum E2 Levels and Uterus Weight and Volume.*

Table S4 One-way analysis of variance (ANOVA) test of *serum E2 levels and uterus weight and volume.*

**Group comparison**	**Statistical analysis**	
**Serum E2 Levels**	***P***	**F ($DF_{between\ group}$, $DF_{residual}$)**
ANOVA followed by Tukey's post hoc test		F (4,20) = 83.586
All group	< 0.001	
Sham vs. OVX + vehicle group	< 0.001	
OVX + vehicle group vs. OVX + 17β-estradiol	< 0.001	
OVX + vehicle group vs. OVX + TKM50	0.856	
OVX + vehicle group vs. OVX + TKM100	0.810	
**Uterus weight**	***P***	**F ($DF_{between\ group}$, $DF_{residual}$)**
ANOVA followed by Tukey's post hoc test		F (4,45) = 1075.252
All group	< 0.001	
Sham vs. OVX + vehicle group	< 0.001	
OVX + vehicle group vs. OVX + 17β-estradiol	< 0.001	
OVX + vehicle group vs. OVX + TKM50	0.834	
OVX + vehicle group vs. OVX + TKM100	0.244	
**Uterus volume**	***P***	**F ($DF_{between\ group}$, $DF_{residual}$)**
ANOVA followed by Tukey's post hoc test		F (4,45) = 518.212
All group	< 0.001	
Sham vs. OVX + vehicle group	< 0.001	
OVX + vehicle group vs. OVX + 17β-estradiol	< 0.001	
OVX + vehicle group vs. OVX + TKM50	0.999	
OVX + vehicle group vs. OVX + TKM100	0.817	

5. *Statistical Analysis Effect of TKM Extract on OVX-Changed Lipid Peroxidation in Hippocampus and Frontal cortex.*

Table S5 One-way analysis of variance (ANOVA) test of lipid peroxidation in hippocampus and frontal cortex.

**Hippocampus**		
**Group comparison**	**Statistical analysis**	
	P	**F ($DF_{between\ group}$, $DF_{residual}$)**
ANOVA followed by Tukey's post hoc test		F (4,20) = 69.023
All group	< 0.001	
Sham vs. OVX + vehicle group	< 0.001	
OVX + vehicle group vs. OVX + 17β-estradiol	< 0.001	
OVX + vehicle group vs. OVX + TKM50	0.064	
OVX + vehicle group vs. OVX + TKM100	< 0.001	
OVX + TKM50 vs. OVX + TKM100	< 0.001	
**Frontal cortex**		
**Group comparison**	**Statistical analysis**	
	P	**F ($DF_{between\ group}$, $DF_{residual}$)**
ANOVA followed by Tukey's post hoc test		F (4,20) = 206.371
All group	< 0.001	
Sham vs. OVX + vehicle group	< 0.001	
OVX + vehicle group vs. OVX + 17β-estradiol	< 0.001	
OVX + vehicle group vs. OVX + TKM50	0.462	
OVX + vehicle group vs. OVX + TKM100	< 0.001	
OVX + TKM50 vs. OVX + TKM100	< 0.001	

6. *Statistical Analysis Effect of TKM Extract on OVX-Changed Antioxidant enzymes Activities in Hippocampus and Frontal cortex.*

Table S6.1 One-way analysis of variance (ANOVA) test of SOD activity in hippocampus and frontal cortex.

**Hippocampus**		
**Group comparison**	**Statistical analysis**	
	P	**F ($DF_{between\ group}$, $DF_{residual}$)**
ANOVA followed by Tukey's post hoc test		F (4,20) = 1000.588
All group	< 0.001	
Sham vs. OVX + vehicle group	< 0.001	
OVX + vehicle group vs. OVX + 17β-estradiol	< 0.001	
OVX + vehicle group vs. OVX + TKM50	0.975	
OVX + vehicle group vs. OVX + TKM100	< 0.001	
OVX + TKM50 vs. OVX + TKM100	< 0.001	

**Frontal cortex**		
**Group comparison**	**Statistical analysis**	
	P	**F ($DF_{between\ group}$, $DF_{residual}$)**
ANOVA followed by Tukey's post hoc test		F (4,20) = 959.920
All group	< 0.001	
Sham vs. OVX + vehicle group	< 0.001	
OVX + vehicle group vs. OVX + 17β-estradiol	< 0.001	
OVX + vehicle group vs. OVX + TKM50	1.000	
OVX + vehicle group vs. OVX + TKM100	< 0.001	
OVX + TKM50 vs. OVX + TKM100	< 0.001	

Table S6.2 One-way analysis of variance (ANOVA) test of CAT activity in hippocampus and frontal cortex.

**Hippocampus**		
**Group comparison**	**Statistical analysis**	
	P	**F ($DF_{between\ group}$, $DF_{residual}$)**
ANOVA followed by Tukey's post hoc test		F (4,20) = 40.898
All group	< 0.001	
Sham vs. OVX + vehicle group	< 0.001	
OVX + vehicle group vs. OVX + 17β-estradiol	< 0.001	
OVX + vehicle group vs. OVX + TKM50	0.912	
OVX + vehicle group vs. OVX + TKM100	< 0.001	
OVX + TKM50 vs. OVX + TKM100	< 0.001	

**Frontal cortex**		
**Group comparison**	**Statistical analysis**	
	P	**F ($DF_{between\ group}$, $DF_{residual}$)**
ANOVA followed by Tukey's post hoc test		F (4,20) = 186.010
All group	< 0.001	
Sham vs. OVX + vehicle group	< 0.001	
OVX + vehicle group vs. OVX + 17β-estradiol	< 0.001	
OVX + vehicle group vs. OVX + TKM50	0.307	
OVX + vehicle group vs. OVX + TKM100	< 0.001	
OVX + TKM50 vs. OVX + TKM100	< 0.001	

7. Statistical Analysis Effect of TKM Extract on OVX-Changed genes expression in Hippocampus and Frontal cortex.

Table S7.1 One-way analysis of variance (ANOVA) test of *ERα* expression in hippocampus and frontal cortex.

**Hippocampus**		
**Group comparison**	**Statistical analysis**	
	P	**F ($DF_{between\ group}$, $DF_{residual}$)**
ANOVA followed by Tukey's post hoc test		F (4,20) = 23.367
All group	< 0.001	
Sham vs. OVX + vehicle group	< 0.001	
OVX + vehicle group vs. OVX + 17β-estradiol	< 0.001	
OVX + vehicle group vs. OVX + TKM50	0.291	
OVX + vehicle group vs. OVX + TKM100	< 0.001	
OVX + TKM50 vs. OVX + TKM100	0.007	
**Frontal cortex**		
**Group comparison**	**Statistical analysis**	
	P	**F ($DF_{between\ group}$, $DF_{residual}$)**
ANOVA followed by Tukey's post hoc test		F (4,20) = 37.870
All group	< 0.001	
Sham vs. OVX + vehicle group	< 0.001	
OVX + vehicle group vs. OVX + 17β-estradiol	< 0.001	
OVX + vehicle group vs. OVX + TKM50	0.891	
OVX + vehicle group vs. OVX + TKM100	< 0.001	
OVX + TKM50 vs. OVX + TKM100	0.010	

Table S7.2 One-way analysis of variance (ANOVA) test of *ERβ* expression in hippocampus and frontal cortex.

**Hippocampus**		
**Group comparison**	**Statistical analysis**	
	P	**F ($DF_{between\ group}$, $DF_{residual}$)**
ANOVA followed by Tukey's post hoc test		F (4,20) = 95.263
All group	< 0.001	
Sham vs. OVX + vehicle group	< 0.001	
OVX + vehicle group vs. OVX + 17β-estradiol	< 0.001	
OVX + vehicle group vs. OVX + TKM50	0.315	
OVX + vehicle group vs. OVX + TKM100	< 0.001	
OVX + TKM50 vs. OVX + TKM100	0.014	
**Frontal cortex**		
**Group comparison**	**Statistical analysis**	
	P	**F ($DF_{between\ group}$, $DF_{residual}$)**
ANOVA followed by Tukey's post hoc test		F (4,20) = 69.020
All group	< 0.001	
Sham vs. OVX + vehicle group	< 0.001	
OVX + vehicle group vs. OVX + 17β-estradiol	< 0.001	
OVX + vehicle group vs. OVX + TKM50	0.715	
OVX + vehicle group vs. OVX + TKM100	0.001	
OVX + TKM50 vs. OVX + TKM100	0.034	

Table S7.3 One-way analysis of variance (ANOVA) test of *Nrf2* expression in hippocampus and frontal cortex.

**Hippocampus**		
**Group comparison**	**Statistical analysis**	
	P	**F ($DF_{between\ group}$, $DF_{residual}$)**
ANOVA followed by Tukey's post hoc test		F (4,20) = 73.192
All group	< 0.001	
Sham vs. OVX + vehicle group	< 0.001	
OVX + vehicle group vs. OVX + 17β-estradiol	< 0.001	
OVX + vehicle group vs. OVX + TKM50	0.130	
OVX + vehicle group vs. OVX + TKM100	< 0.001	
OVX + TKM50 vs. OVX + TKM100	< 0.001	
**Frontal cortex**		
**Group comparison**	**Statistical analysis**	
	P	**F ($DF_{between\ group}$, $DF_{residual}$)**
ANOVA followed by Tukey's post hoc test		F (4,20) = 50.320
All group	< 0.001	
Sham vs. OVX + vehicle group	< 0.001	
OVX + vehicle group vs. OVX + 17β-estradiol	< 0.001	
OVX + vehicle group vs. OVX + TKM50	0.959	
OVX + vehicle group vs. OVX + TKM100	< 0.001	
OVX + TKM50 vs. OVX + TKM100	< 0.001	

Table S7.4 One-way analysis of variance (ANOVA) test of *Keap1* expression in hippocampus and frontal cortex.

**Hippocampus**		
**Group comparison**	**Statistical analysis**	
	P	**F ($DF_{between\ group}$, $DF_{residual}$)**
ANOVA followed by Tukey's post hoc test		F (4,20) = 31.271
All group	< 0.001	
Sham vs. OVX + vehicle group	< 0.001	
OVX + vehicle group vs. OVX + 17β-estradiol	< 0.001	
OVX + vehicle group vs. OVX + TKM50	0.714	
OVX + vehicle group vs. OVX + TKM100	< 0.001	
OVX + TKM50 vs. OVX + TKM100	0.002	
**Frontal cortex**		
**Group comparison**	**Statistical analysis**	
	P	**F ($DF_{between\ group}$, $DF_{residual}$)**
ANOVA followed by Tukey's post hoc test		F (4,20) = 38.000
All group	< 0.001	
Sham vs. OVX + vehicle group	< 0.001	
OVX + vehicle group vs. OVX + 17β-estradiol	< 0.001	
OVX + vehicle group vs. OVX + TKM50	0.491	
OVX + vehicle group vs. OVX + TKM100	< 0.001	
OVX + TKM50 vs. OVX + TKM100	0.027	

Table S7.5 One-way analysis of variance (ANOVA) test of *BDNF* expression in hippocampus and frontal cortex.

**Hippocampus**		
**Group comparison**	**Statistical analysis**	
	P	**F ($DF_{between\ group}$, $DF_{residual}$)**
ANOVA followed by Tukey's post hoc test		
All group	< 0.001	F (4,20) = 174.943
Sham vs. OVX + vehicle group	< 0.001	
OVX + vehicle group vs. OVX + 17β-estradiol	< 0.001	
OVX + vehicle group vs. OVX + TKM50	0.609	
OVX + vehicle group vs. OVX + TKM100	< 0.001	
OVX + TKM50 vs. OVX + TKM100	< 0.001	
**Frontal cortex**		
**Group comparison**	**Statistical analysis**	
	P	**F ($DF_{between\ group}$, $DF_{residual}$)**
ANOVA followed by Tukey's post hoc test		
All group	< 0.001	F (4,20) = 325.911
Sham vs. OVX + vehicle group	< 0.001	
OVX + vehicle group vs. OVX + 17β-estradiol	< 0.001	
OVX + vehicle group vs. OVX + TKM50	0.956	
OVX + vehicle group vs. OVX + TKM100	< 0.001	
OVX + TKM50 vs. OVX + TKM100	< 0.001	

Table S7.6 One-way analysis of variance (ANOVA) test of *CREB* expression in hippocampus and frontal cortex.

**Hippocampus**		
**Group comparison**	**Statistical analysis**	
	P	**F ($DF_{between\ group}$, $DF_{residual}$)**
ANOVA followed by Tukey's post hoc test		
All group	< 0.001	F (4,20) = 101.167
Sham vs. OVX + vehicle group	< 0.001	
OVX + vehicle group vs. OVX + 17β-estradiol	< 0.001	
OVX + vehicle group vs. OVX + TKM50	0.961	
OVX + vehicle group vs. OVX + TKM100	< 0.001	
OVX + TKM50 vs. OVX + TKM100	< 0.001	
**Frontal cortex**		
**Group comparison**	**Statistical analysis**	
	P	**F ($DF_{between\ group}$, $DF_{residual}$)**
ANOVA followed by Tukey's post hoc test		
All group	< 0.001	F (4,20) = 89.815
Sham vs. OVX + vehicle group	< 0.001	
OVX + vehicle group vs. OVX + 17β-estradiol	< 0.001	
OVX + vehicle group vs. OVX + TKM50	0.878	
OVX + vehicle group vs. OVX + TKM100	< 0.001	
OVX + TKM50 vs. OVX + TKM100	< 0.001	

Table S8. The percentage yield of Tri-Kaysorn-Mas (TKM) extract and its herbal components

Ethanolic extraction (maceration method)	Powder (g)	Solvent (L) ratio 1:4 (w/v)	Crude extract (g)	%yield (w/w)
Tri-Kaysorn-Mas	2700	10800	290.77	10.77
Aegle marmelos L.	800	3200	92.19	11.52
Jatropha multifida L.	800	3200	49.27	6.16
Nelumbo nucifera Geartn.	800	3200	92.22	11.53

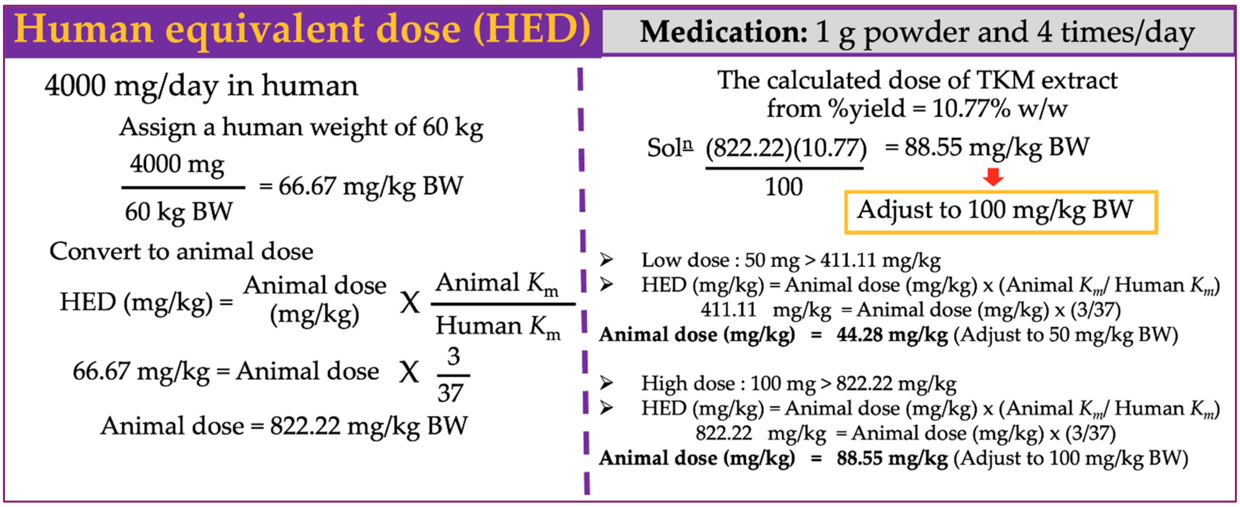

Figure S1. The calculated dose of Tri-Kaysorn-Mas (TKM) extract

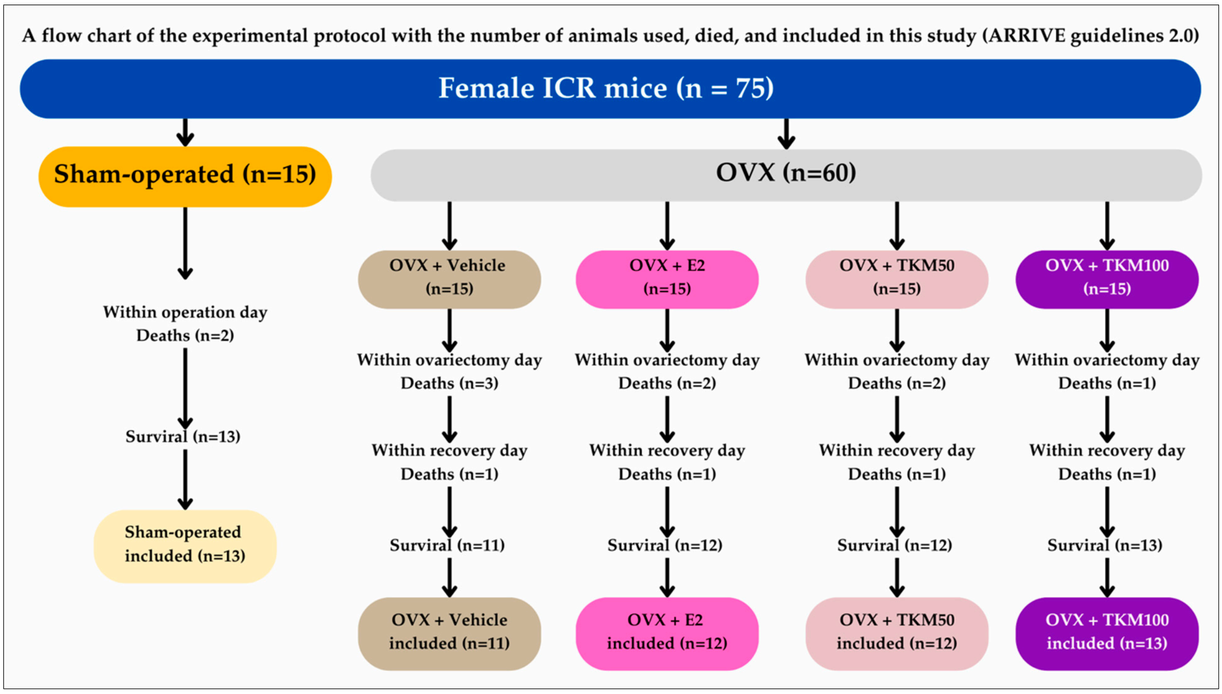

Figure S2. The ARRIVE guidelines 2.0 of Tri-Kaysorn-Mas (TKM) extract
